# Prophages and Growth Dynamics Confound Experimental Results with Antibiotic-Tolerant Persister Cells

**DOI:** 10.1128/mBio.01964-17

**Published:** 2017-12-12

**Authors:** Alexander Harms, Cinzia Fino, Michael A. Sørensen, Szabolcs Semsey, Kenn Gerdes

**Affiliations:** Centre of Excellence for Bacterial Stress Response and Persistence, Department of Biology, University of Copenhagen, Copenhagen, Denmark; University of Würzburg

**Keywords:** (p)ppGpp, antibiotic tolerance, bacteriophage genetics, persistence, toxin-antitoxin modules

## Abstract

Bacterial persisters are phenotypic variants that survive antibiotic treatment in a dormant state and can be formed by multiple pathways. We recently proposed that the second messenger (p)ppGpp drives *Escherichia coli* persister formation through protease Lon and activation of toxin-antitoxin (TA) modules. This model found considerable support among researchers studying persisters but also generated controversy as part of recent debates in the field. In this study, we therefore used our previous work as a model to critically examine common experimental procedures to understand and overcome the inconsistencies often observed between results of different laboratories. Our results show that seemingly simple antibiotic killing assays are very sensitive to variations in culture conditions and bacterial growth phase. Additionally, we found that some assay conditions cause the killing of antibiotic-tolerant persisters via induction of cryptic prophages. Similarly, the inadvertent infection of mutant strains with bacteriophage ϕ80, a notorious laboratory contaminant, apparently caused several of the phenotypes that we reported in our previous studies. We therefore reconstructed all infected mutants and probed the validity of our model of persister formation in a refined assay setup that uses robust culture conditions and unravels the dynamics of persister cells through all bacterial growth stages. Our results confirm the importance of (p)ppGpp and Lon but no longer support a role of TA modules in *E. coli* persister formation under unstressed conditions. We anticipate that the results and approaches reported in our study will lay the ground for future work in the field.

## IMPORTANCE

The recalcitrance of antibiotic-tolerant persister cells is thought to cause relapsing infections and antibiotic treatment failure in various clinical setups. Previous studies identified multiple genetic pathways involved in persister formation but also revealed reproducibility problems that sparked controversies about adequate tools to study persister cells. In this study, we unraveled how typical antibiotic killing assays often fail to capture the biology of persisters and instead give widely differing results based on poorly controlled experimental parameters and artifacts caused by cryptic as well as contaminant prophages. We therefore established a new, robust assay that enabled us to follow the dynamics of persister cells through all growth stages of bacterial cultures without distortions by bacteriophages. This system also favored adequate comparisons of mutant strains with aberrant growth phenotypes. We anticipate that our results will contribute to a robust, common basis for future studies on the formation and eradication of antibiotic-tolerant persisters.

## INTRODUCTION

Bacterial persisters constitute a subpopulation of phenotypically antibiotic-tolerant cells that form within a population of genetically antibiotic-susceptible bacteria. Persister cells are usually slowly growing or nongrowing, and researchers in the field largely agree that the antibiotic tolerance of persisters is linked to a dormant physiological state in which the cellular processes commonly poisoned by bactericidal antibiotics are inactive (1, 2). Consistently, a large body of studies from several laboratories has uncovered genetic pathways that control and execute the formation of persister cells of *Escherichia coli* K-12 as a phenotypic conversion into dormancy (1, 2). These mechanisms include a drop of cellular ATP levels, the modulation of nucleoid-associated proteins, changes in metabolic fluxes, high expression of drug efflux pumps, or the activation of different sets of toxin-antitoxin (TA) modules (3–10). The emerging picture is therefore that various parallel and partially interlinked pathways of persister formation in *E. coli* K-12 give rise to a heterogeneous population of persisters that have formed through different pathways, have different physiological properties, and thus exhibit different profiles of antibiotic tolerance (1, 2, 11).

The complexity of bacterial persister formation and the sensitivity of persistence assays to even slight experimental variation have generated controversies that have made the field notorious for debates on the technical and biological aspects of studying bacterial persistence (12–16). Among the work cited above, our laboratory has published a series of studies linking persister formation in *E. coli* K-12 to the activation of a set of 10 mRNA endonuclease toxin TA modules under control of the second messenger (p)ppGpp, polyphosphate, and protease Lon (8, 9). Though similar findings have been made, e.g., in *Salmonella enterica* serovar Typhimurium (17), our model has also been met with skepticism by other researchers in the field (5, 13, 14, 18). In the present study, we therefore carefully reevaluated our previous conclusions as well as the underlying methodology and also probed common experimental procedures for sources of the frequently observed inconsistencies between studies in the field.

In short, we discovered that *E. coli* mutant strains used in our previous work had been inadvertently infected with several different lysogenic bacteriophages and that prophage carriage strongly affected persistence measurements. However, we also show that already the resident cryptic prophages of the *E. coli* K-12 MG1655 wild-type strain distort the results of persister assays under conditions that are commonly used in the field. Importantly, we show that the common practice of determining persister levels at only a single growth time point is often inappropriate to account for shifted dynamics of growth and persister formation in mutant strains. We finally tested the key components of our previously proposed model of persister formation using new mutant strains and a refined methodology. Crucially, we could confirm a role of (p)ppGpp, polyphosphate, and Lon in bacterial persister formation and/or survival but did not find strong evidence for the involvement of TA modules or the connection of these components in a single pathway of persister formation under unstressed conditions.

## RESULTS

### Classical persister assays suffer from technical and biological drawbacks.

**FIG 1:**
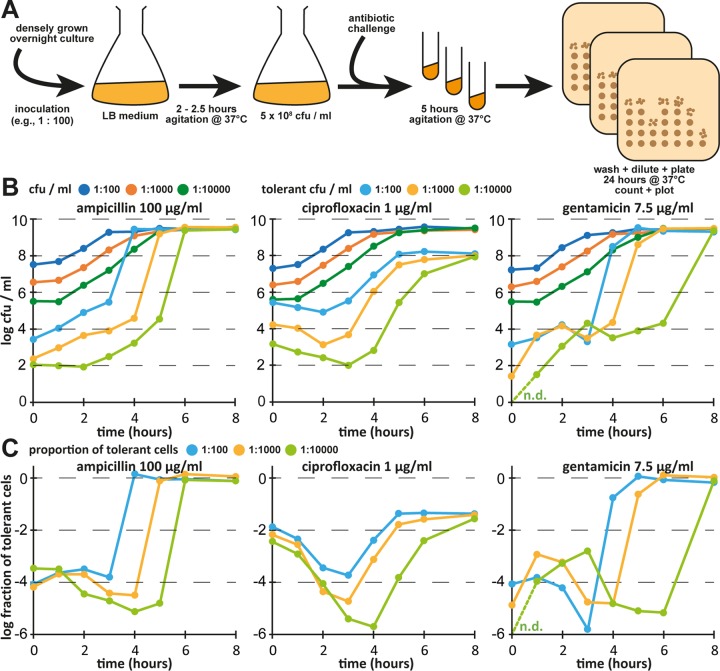
Persister assays are affected by inoculum and growth phase. (A) Scheme illustrating the setup of a persister assay as it is commonly performed in the field. (B) Cultures of *E. coli* K-12 MG1655 were grown in LB medium after inoculation at a dilution of 1:100 (blue), 1:1,000 (orange), or 1:10,000 (green) from dense overnight cultures, and levels of all CFUs as well as levels of antibiotic-tolerant CFUs were determined at each time point. (C) Fraction of antibiotic-tolerant cells for each data point reported as described for panel B. Data points are shown for one representative experiment, because the absolute numbers of antibiotic-tolerant cells (but not their dynamics) were affected considerably by batch-to-batch variations of the LB medium (see Fig. S1). n.d., not detected (no gentamicin-tolerant bacteria recovered at *t* = 0 h from inoculation at a dilution of 1:10,000).

The formation of persister cells is typically measured by determining the fraction of antibiotic-tolerant cells in bacterial cultures that are considered to be exponentially growing some hours after inoculation from dense overnight cultures (Fig. 1A). A biphasic kinetic of antibiotic killing reveals the presence of persister cells, because these are killed slowly and can be detected after the regular cells have been rapidly eliminated in a first phase of killing (19). Despite the apparent simplicity of this experimental setup, persister assays are known to be sensitive to even minor variations of the experimental conditions and have often given inconsistent results in different laboratories (5, 16, 20, 21). We therefore suspected that this simple assay setup may be inadequate to represent the dynamic nature of bacterial persistence and could give results that are strongly affected by biological or technical parameters that are usually not controlled in persistence assays.

10.1128/mBio.01964-17.1 FIG S1  The experiment whose results are shown in Fig. 1B and C was performed with a different batch of LB medium. We performed the experiment whose results are shown in Fig. 1B and C with a different batch of LB medium and compared CFU counts per milliliter (A) as well as the fractions of antibiotic-tolerant cells (B) after treatment with ciprofloxacin at 1 µg/ml. Note that the growth rates seen with regular bacteria were not different between batch 1 (shown in Fig. 1) and batch 2 (shown here) but that the dynamics of persister levels differed at early time points. Download FIG S1, PDF file, 0.5 MB. Copyright © 2017 Harms et al. 2017 Harms et al. This content is distributed under the terms of the Creative Commons Attribution 4.0 International license.

As a first step toward understanding the notorious variability of results reported for single-growth-time-point persister assays, we followed the absolute levels of colony forming units (CFUs) and antibiotic-tolerant cells over time from inoculation of an *E. coli* culture through exponential growth into stationary phase in Luria-Bertani (LB) medium (Fig. 1B and C). Cultures were treated with lethal concentrations of ampicillin, ciprofloxacin, or gentamicin (representing β-lactams, fluoroquinolones, and aminoglycosides that kill by very different mechanisms) for 5 h that are more than sufficient to kill all regular cells at least during exponential growth so that only persisters remain (5, 8, 9, 11).

Most importantly, we found that cultures inoculated with different numbers of cells from overnight cultures displayed systematically different fractions of antibiotic-tolerant cells at any given total CFU count per milliliter during exponential growth (e.g., at ca. 5 × 10^8^ CFU/ml reached after 2, 3, and 4 h of growth under conditions of inoculation at 1:100, 1:1,000, and 1:10,000 dilutions from stationary-phase overnight cultures, respectively; Fig. 1B and C). As an example, the fraction of cells tolerant to 1 µg/ml of ciprofloxacin, a commonly used treatment setup, was 10^−4^, 10^−5^, or 10^−6^ at this growth stage (5 × 10^8^ CFU/ml) depending on the inoculum. Similar findings had already been published previously by Balaban et al. (22) but were not widely taken into account for the design and interpretation of antibiotic killing assays. Importantly, these observations are incompatible with the idea that this setup of persister assays is measuring stochastic persister formation of exponentially growing bacteria, since this should be independent of the inoculum. Furthermore, the levels of tolerant cells differed massively between the three antibiotics and over time even during periods when the overall CFU per milliliter changed only marginally, highlighting that the heterogeneity of persister cells makes it inappropriate to simply report “persister levels” from a single time point or a given cell density.

In addition, we observed variations of results obtained with different batches of LB medium in a manner in which bacterial growth and the overall dynamics of antibiotic tolerance were not affected but in which the absolute levels of tolerant cells varied considerably (see Fig. S1 in the supplemental material). It is well known that LB medium is prone to batch-to-batch variation that essentially cannot be controlled in the laboratory, e.g., due to the degradation of l-tryptophan over time depending on the exposure to ambient light or the degree of l-asparagine and l-glutamine deamidation during autoclaving (23). Furthermore, the poor content of divalent cations and sugars in LB medium causes variations in the availability of these important nutrients to *E. coli* cultures due to even minor differences in handling during the preparation of each batch (24).

### Following the dynamics of *E. coli* persister cells in M9 medium.

**FIG 2:**
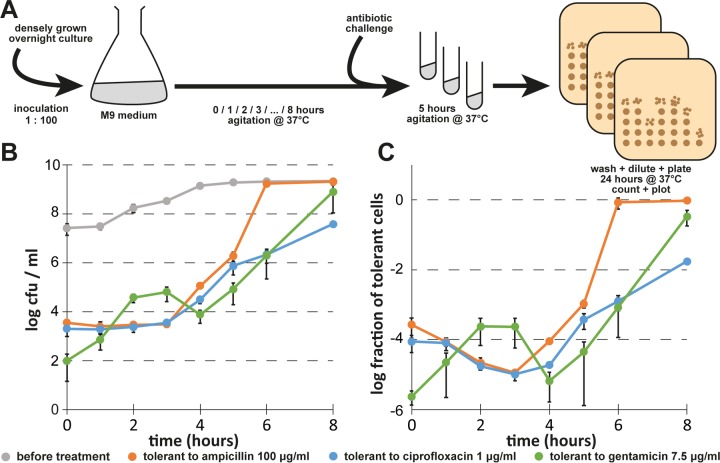
Persister formation of *E. coli* K-12 MG1655 in M9 medium. (A) Scheme illustrating how the dynamics of persister formation were determined. (B) Bacterial growth and the dynamics of antibiotic-tolerant cells were determined for cultures of *E. coli* K-12 MG1655 in M9 minimal medium as described for the experiment whose results are shown in Fig. 1 after inoculation at a dilution of 1:100 from a dense overnight culture. (C) Fraction of antibiotic-tolerant cells for each data point shown in panel B. We generated biphasic kill curves to verify that the antibiotic-tolerant cells present during exponential growth (3 h after inoculation) represented persisters (Fig. S2). Data points represent means of results from at least three independent experiments, and error bars indicate standard deviations.

To overcome the issues outlined above, we decided to switch the culture medium from LB to the more defined M9 medium (see details in Materials and Methods) and adopted the determination of antibiotic tolerance in bacterial cultures over time as our standard procedure (Fig. 2A). Studying the dynamics of *E. coli* K-12 wild-type cells tolerant to ampicillin, ciprofloxacin, or gentamicin as described above, the results of experimental replicates were much more homogeneous and showed similarities to but also differences from those obtained in previous experiments performed in LB medium (Fig. 2B and C). For ampicillin, the level of tolerant cells remained constant after inoculation throughout exponential growth and then sharply increased during late-exponential growth until all bacteria were ampicillin-tolerant in stationary phase, because this antibiotic is unable to kill nongrowing cells (25). Similarly, the number of ciprofloxacin-tolerant cells was stable for the first hours and then increased into stationary phase, while—unlike the results seen with ampicillin—only up to 10% of the population became tolerant. For gentamicin, the dynamics of antibiotic-tolerant cells looked very different from those seen with the other antibiotics, and the levels of tolerant cells initially increased until mid-exponential phase, decreased again until entry into stationary phase, and finally rose to almost full tolerance at the end of the experiment. We interpret these results as indicating that the stable levels of ampicillin- and ciprofloxacin-tolerant cells after inoculation and throughout early exponential growth represent dormant cells that have been carried over from the overnight cultures and not a steady state of persister formation and resuscitation. This interpretation is also in line with the direct correlation of the inoculum with the fraction of antibiotic-tolerant cells that we had observed before (Fig. 1B and C).

10.1128/mBio.01964-17.2 FIG S2  Biphasic killing of exponentially growing *E. coli* K-12 MG1655 in M9 medium. Bacteria were cultured as described for the experiments whose results are shown in Fig. 2 and were treated with 100 µg/ml ampicillin, 1 µg/ml ciprofloxacin, or 7.5 µg/ml gentamicin. Samples were collected at different time points for 5 h, and the number of surviving CFU per milliliter was plotted against the duration of treatment. Note that *E. coli* K-12 displayed biphasic killing when challenged with any of the three antibiotics while growing exponentially in M9 medium, suggesting that tolerance assays at this growth phase report bacterial persister cells. Data points represent means of the results from at least three independent experiments, and error bars indicate standard deviations. Download FIG S2, PDF file, 0.4 MB. Copyright © 2017 Harms et al. 2017 Harms et al. This content is distributed under the terms of the Creative Commons Attribution 4.0 International license.

### Persister assays with ciprofloxacin in *E. coli* K-12 are affected by induction of resident prophages.

**FIG 3:**
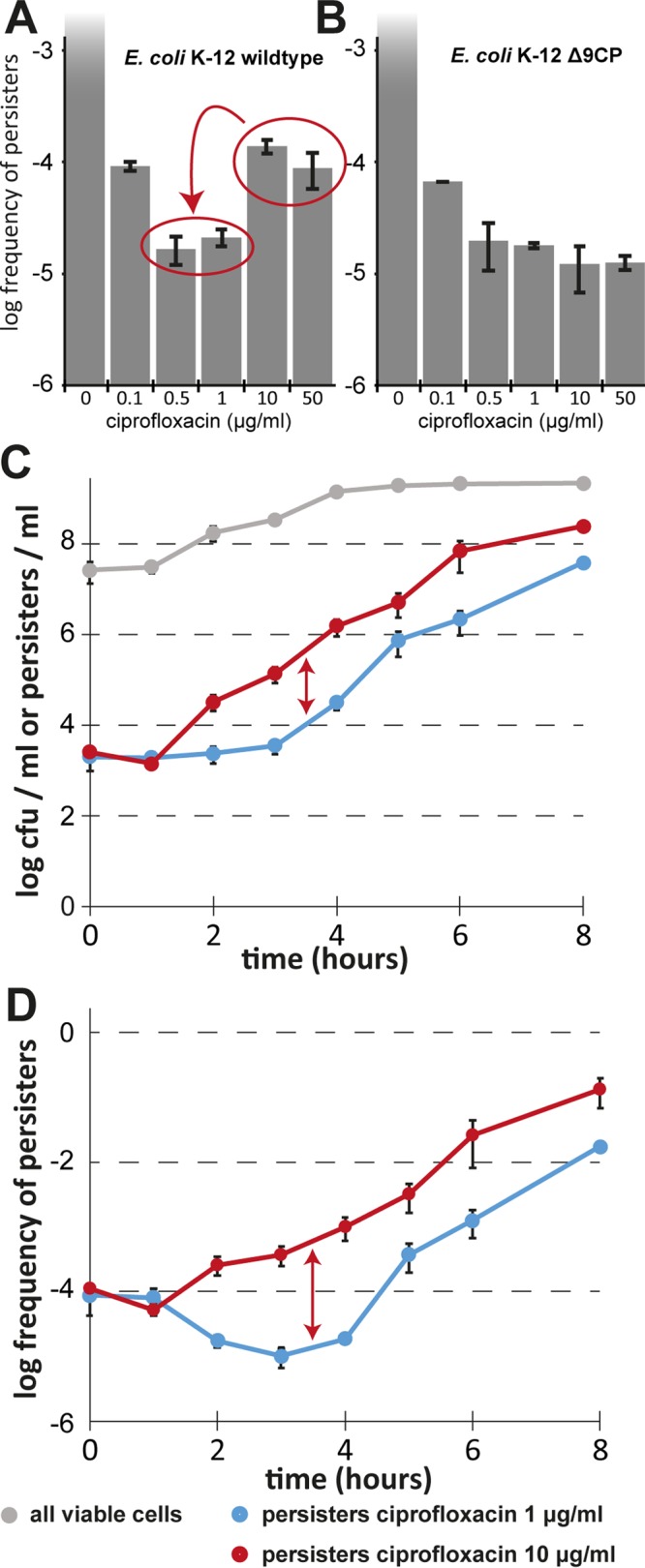
Induction of cryptic prophages distorts persister measurements of *E. coli* K-12. Exponentially growing cultures of *E. coli* K-12 strain BW25113 (A) and its derivative lacking all nine cryptic prophages (B; *Δ*9CP) created by Wang et al. (28) were treated with different concentrations of ciprofloxacin in M9 medium, and the level of surviving persister cells was determined. Note that the level of survivors for the wild-type strain increased at high ciprofloxacin concentrations, while no such effect can be observed in the Δ9CP mutant. The latter strain generally exhibits a lower level of persister formation than its ancestor, possibly due to the roles of prophage-encoded factors in persistence and general stress tolerance (28). (C) Bacterial growth and the dynamics of antibiotic-tolerant cells were determined for cultures of *E. coli* K-12 MG1655 in M9 minimal medium as described for Fig. 2B, and the results of treatment with 1 µg/ml and 10 µg/ml ciprofloxacin were compared. (D) Data representing the fraction of antibiotic-tolerant cells at each time point are shown. Data points represent means of results from at least three independent experiments, and error bars indicate standard deviations.

Beyond these issues related to the growth conditions of bacteria studied in persistence assays, a recent publication by members of the Brynildsen laboratory on *Staphylococcus aureus* persister formation alerted us that the presence of resident prophages might affect measurements of persister frequencies under some conditions (26). In short, those authors showed that ciprofloxacin treatment at the commonly used concentrations of 0.5 to 1 µg/ml—more than an order of magnitude above the MIC and sufficient to kill all regular cells—eliminates a substantial fraction of persisters and that the effect is not due to the direct action of the antibiotic but is instead due to the secondary activation of prophages. This effect could be overcome at very high concentrations of ciprofloxacin that cause a strong inhibition of cellular DNA processing and thus impair prophage development (26). Given that DNA damage is generally known to be a strong inducer of temperate prophages, including some of the cryptic prophages in the *E. coli* K-12 MG1655 chromosome (27, 28), we suspected that a similar effect described by Sandvik et al. (26) may also affect the persister results reported for *E. coli*. We therefore compared the *E. coli* K-12 wild-type strain to a previously published mutant devoid of all nine cryptic prophages for tolerance to different concentrations of ciprofloxacin in a classical single-growth-time-point persister assay (Fig. 3). Similarly to the results reported for *S. aureus*, the *E. coli* K-12 wild-type strain showed a minimum of survival at commonly used intermediate concentrations of ciprofloxacin (0.5 to 1 µg/ml) and displayed greatly increased survival at higher concentrations (Fig. 3A). This effect was abolished in the mutant lacking the cryptic prophages (Fig. 3B). Studying the gap between survival of 1 µg/ml ciprofloxacin treatment and survival of 10 µg/ml ciprofloxacin treatment over time, we found that the difference was considerable (around 1 log) throughout all growth phases but was most pronounced during exponential growth (Fig. 3C and D). To avoid artifacts from the induction of cryptic prophages, we therefore adopted 10 µg/ml as the standard concentration of ciprofloxacin for antibiotic killing assays.

### *E. coli Δ1-10TA* bacteria carry a defective lambda prophage.

The major effect of cryptic prophages on the detection of ciprofloxacin-tolerant persisters caught our attention, because a previous analysis of the genome sequence of our *E. coli* K-12 MG1655 *Δ10TA* strain by the members of Kim Lewis’s laboratory had revealed an accumulation of nucleotide polymorphisms in the cryptic prophages (5). The *Δ10TA* strain had been created by the sequential deletion of 10 mRNA endonuclease toxin TA modules and was described to display the strong defect in bacterial persistence that was a cornerstone of our previous work (8, 9). We recently discovered that the published variant of *E. coli Δ10TA* carried a lambda prophage. The infection was easily cured genetically (see Materials and Methods), and the resulting strain, *E. coli* K-12 MG1655 *Δ10TA attB*(*+*), did not show any difference from parental *E. coli* K-12 MG1655 *Δ10TA* λ(*+*) in persister levels (see below). However, a general concern regarding prophage-mediated effects on persistence and the sustained debate over the results of our previous work prompted us to sequence the genome of *E. coli* K-12 MG1655 *Δ10TA attB*(*+*). Additionally, we sequenced two ancestors of mutant *Δ10TA*, namely, strains *Δ5TA* and *Δ8TA*, in which five and eight mRNA endonuclease toxin TA modules had been deleted, respectively (9). Notably, the *Δ5TA* mutant had been the first strain in the series of sequential TA module deletions of our previous work that was found to exhibit a defect in persister formation (9).

**FIG 4:**
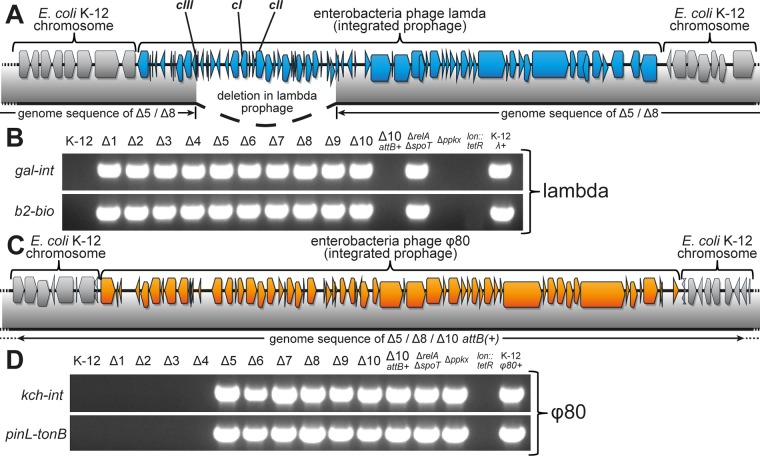
Lambda and ϕ80 infection of strains from our previous studies. (A) The insertion of a lambda prophage (blue gene arrows) in the *E. coli* K-12 chromosome (gray gene arrows) is shown together with coverage of the prophage insertion in the genomes of the *Δ5TA* and *Δ8TA* mutants (gray bar below). The 13-kilobase deletion comprising *cI*, *cII*, and *cIII* repressors spans a sequence from the *gam* nuclease inhibitor gene to the endolysin gene *R*. (B) Diagnostic PCR analyses of the two junctions between the lambda prophage and the *gal-bio* region of the *E. coli* chromosome were performed to determine the extent of lambda infection in important strains from our previous studies (8, 9). (C) Insertion of a ϕ80 prophage (orange gene arrows) in the *E. coli* K-12 chromosome (gray gene arrows) in the genomes of *Δ5TA*, *Δ8TA*, and *Δ10TA attB*(*+*) mutants. (D) Diagnostic PCR analyses of the two junctions between the ϕ80 prophage and the *yciI* locus in the *E. coli* K-12 chromosome were performed to determine the prevalence of ϕ80 infections among important strains from our previous work (8, 9).

Surprisingly, the genome sequences showed that mutants *Δ5TA* and *Δ8TA* carried a lambda prophage at the *attB* site in the *gal-bio* region of the *E. coli* chromosome (Fig. 4A). We therefore tested the most important strains of our previous studies for lambda infection by PCR and found that every TA module deletion strain of the series leading to mutant *Δ10TA*, as well as the *relA spoT* mutant [deficient in (p)ppGpp signaling], was a lambda lysogen (Fig. 4B). Interestingly, the genome sequences of mutants *Δ5TA* and *Δ8TA* revealed a deletion of ca. 13 kb inside the lambda prophage around the major repressor genes *cI*, *cII*, and *cIII* and major promoters, suggesting that the prophage is defective and largely inactive (Fig. 4A).

### Widespread infection with ϕ80 in strains used to support the model.

Beyond a defective lambda prophage in mutants *Δ5TA* and *Δ8TA*, we discovered that each of the three sequenced strains of our TA module deletion series carried a ϕ80 prophage at its *attP* integration site in the *yciI* gene (Fig. 4C). We therefore performed diagnostic PCRs on the junctions of ϕ80 integration in all strains that were crucial for the main results of our previous articles (8, 9). These experiments revealed ϕ80 infections in the *Δ5TA*-*Δ10TA*, *relA spoT*, and *ppkx* (polyphosphate metabolism) mutants (Fig. 4D).

**FIG 5:**
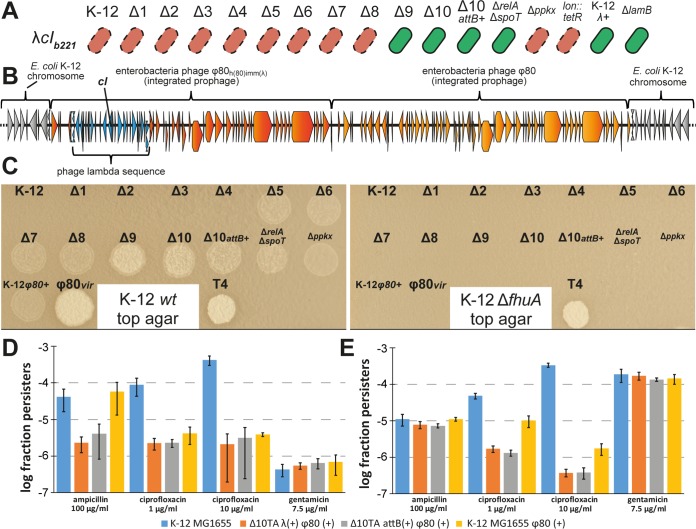
Lambda immunity and active ϕ80 prophages and their effect on persistence. (A) The sensitivity of different *E. coli* strains to the lambda *cI*_*b221*_ mutant was determined by streaking across lines of phage stock on agar plates. Red, no growth/sensitive; green, growth/immune. (B) Illustration of neighboring ϕ80 and ϕ80_*h*(*80*)*imm*(*λ*)_ integration in the chromosome of *E. coli Δ10TA* as deduced from the genome sequence. The *E. coli* chromosome backbone is shown in gray; genes of ϕ80 and ϕ80_*h*(*80*)*imm*(*λ*)_ are shown in orange and dark orange, respectively; and the lambda region of the ϕ80_*h*(*80*)*imm*(*λ*)_ mutant is highlighted in blue. Note that the genome sequence does not unambiguously tell which of the prophages is upstream and which is downstream in the integration site. (C) A plaque assay with culture supernatants and control phage stocks was performed to determine the infectivity of prophage carriers. *wt*, wild type. (D and E) Exponentially growing *E. coli* K-12 MG1655 and different mutant derivatives/lysogens were challenged with ampicillin, ciprofloxacin, or gentamicin in LB medium (D) or M9 medium (E), and the fractions of surviving persisters were calculated. Data points represent averages of results from three independent experiments, and error bars represent standard deviations.

In parallel to studying the ϕ80 infections, we investigated whether the lambda prophages in our strains all carried the 13-kb deletion revealed for mutants *Δ5TA* and *Δ8TA* by testing their susceptibility to the lambda *cI*_*b221*_ mutant encoding an inactive *cI* repressor gene. The lambda *cI*_*b221*_ mutant is obligately lytic unless *cI* is provided in *trans*, e.g., by a lambda prophage. Surprisingly, the *Δ10TA attB*(*+*) strain was immune to lambda *cI*_*b221*_ infection although it had been cured of the lambda prophage, but all TA module deletion strains before and including mutant *Δ8TA* were sensitive (Fig. 5A). These results show that the lambda prophage had already been inactivated in mutant *Δ1TA* and may already have been present in the ancestral *E. coli* K-12 MG1655 wild-type stock. The surprising immunity of mutants *Δ9TA* and *Δ10TA* to lambda *cI*_*b221*_ suggested that these strains must encode a source of lambda *cI* outside the defective prophage that lacks *cI*. Consistently, a deeper analysis of the *Δ10TA attB*(*+*) genome sequence identified a 12-kb piece of lambda centered around the *cI* gene (Fig. 5B). Interestingly, the flanking regions of this lambda segment were ϕ80 sequences and not part of the *E. coli* K-12 MG1655 genome, indicating that these strains had been infected with a ϕ80/lambda hybrid on top of the ϕ80 wild-type phage. Given that the genetic architectures of these two phages are very similar, hybrid phages are often viable and had been used as tools to study different features of phage biology in earlier times (29). A comparison of the *Δ10TA attB*(*+*) genome sequence with those of the published ϕ80/lambda hybrids revealed that the hybrid phage in our strains is known as ϕ80_*h*(*80*)*imm*(*λ*)_, a phage that produces ϕ80 particles but carries a lambda immunity region (Fig. 5B) (29).

Unlike the lambda prophage, the ϕ80 prophages seemed complete and thus likely active. This observation caught our attention, because ϕ80 is known as a highly infectious laboratory contaminant (30, 31) and because we had widely distributed the strains of our TA module deletion series to other research groups. We therefore performed plaque assays on regular overnight cultures of various strains to determine the infectivity of ϕ80 present in our lysogens. As expected, culture supernatants of all strains that had been found to carry a ϕ80 prophage by PCR (Fig. 4C) formed plaques on a lawn of *E. coli* K-12 wild-type cells but did not do so on cells of a *fhuA* mutant that lacks the ϕ80 receptor (Fig. 5C).

### Lysogenization with ϕ80 reduces persisters to levels similar to those seen with TA module deletions in mutant *Δ10TA.*

We were particularly concerned by the ϕ80 lysogenization because this prophage is known to be easily induced by DNA damage (30) and because this property might reduce the number of survivors of treatment with ciprofloxacin, the major antibiotic that was used in our previous study (8). We therefore lysogenized the *E. coli* K-12 MG1655 wild-type strain using ϕ80 particles from the supernatant of mutant *Δ10TA attB*(*+*) and assayed persister formation of this strain in direct comparison to the levels seen with the parental wild-type strain and the *Δ10TA* mutant (Fig. 5D). When the experiment was performed as a single-growth-time-point assay in LB medium as in our previous work, we readily reproduced the significant drop of ampicillin and ciprofloxacin survival that had been reported for the *Δ10TA* mutants compared to the *E. coli* K-12 wild-type strain (8, 9). Lysogenization of *E. coli* K-12 with ϕ80 had no effect on ampicillin tolerance but caused the same drop of ciprofloxacin survival that had been a leading phenotype of mutant *Δ10TA* and other infected strains in our previous studies (Fig. 5D) (8, 9). Furthermore, the difference between the *E. coli* K-12 wild-type strain and the *Δ10TA* mutants in ampicillin tolerance disappeared when the experiment was performed in M9 minimal medium (Fig. 5E), as had already been reported by Shan et al. (5). Taken together, these results directly questioned whether there was any ϕ80-independent difference between the *E. coli* K-12 wild-type strain and the *Δ10TA* mutants in persister formation or survival. In M9 minimal medium, interestingly, the *Δ10TA* strains displayed clearly reduced survival of ciprofloxacin treatment compared to the results seen with the ϕ80 lysogen, possibly due to some of the additional mutations present in the *Δ10TA* genome revealed by Shan et al. (5) or due to the additional presence of ϕ80_*h*(*80*)*imm*(*λ*)_. Unlike what we had observed for the cryptic prophages of *E. coli* K-12 (Fig. 3), an increase in the ciprofloxacin concentration from the commonly used concentration of 1 µg/ml to 10 µg/ml did not reduce the drop in survival caused by lysogenization with ϕ80 (Fig. 5D and E). It seems likely that this difference in behavior was due to the high sensitivity of ϕ80 induction to even slight DNA damage that may remain after the end of ciprofloxacin treatment and persister resuscitation (30), while the induction of cryptic prophages of *E. coli* K-12 requires very strong DNA damage (28). No differences between the *Δ10TA* mutants with and without the defective lambda prophage in the *attB* site or between any strains regarding gentamicin tolerance could be detected (Fig. 5D and E).

### ppGpp, Lon, and polyphosphate in *E. coli* K-12 persister formation.

**FIG 6:**
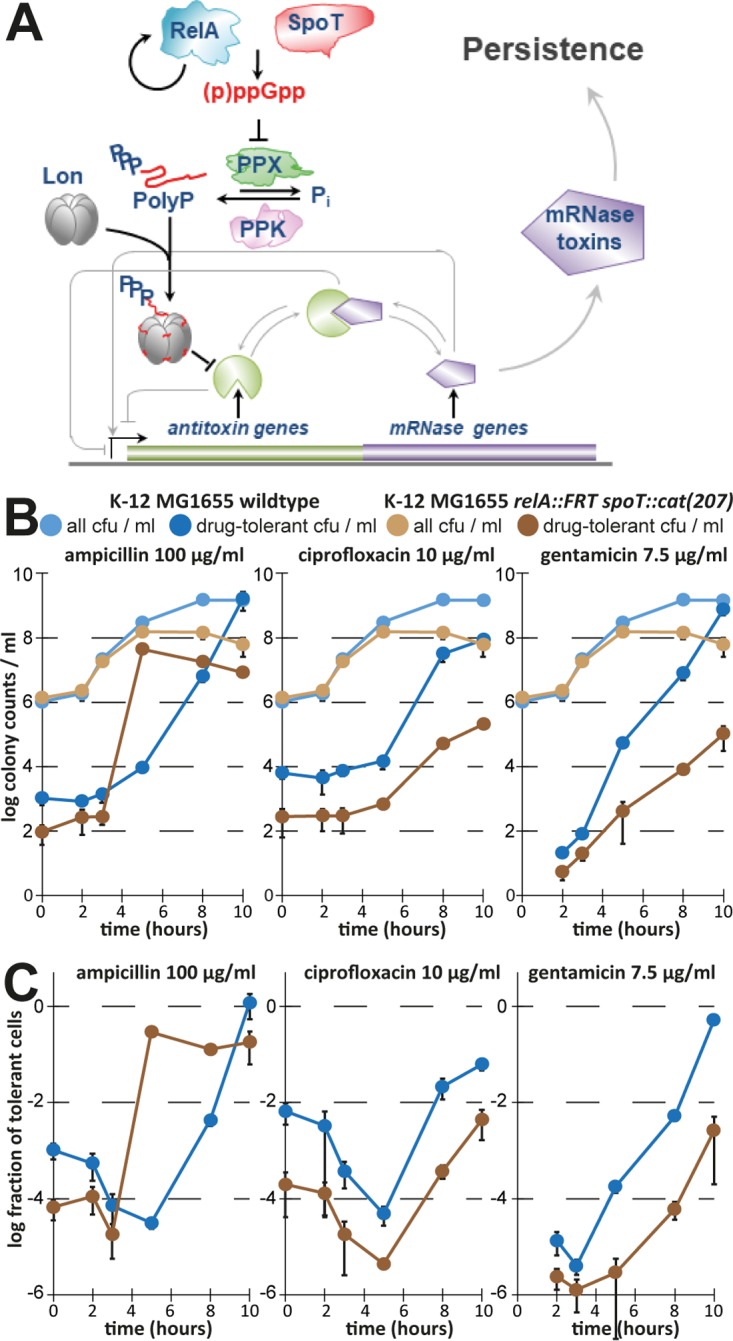
The model of (p)ppGpp-dependent persister formation through TA modules. (A) The illustration (adapted from Germain et al. [54]) shows our previously published model of persister formation initiated by stochastic bursts of (p)ppGpp that induce the production of polyphosphate which stimulates Lon to degrade TA module antitoxins. Consequently, the activation of mRNA interferase toxins would induce bacterial persistence (8). (B and C) In order to verify the most upstream element of the model, we created a new (p)ppGpp-deficient mutant of *E. coli* K-12 MG1655 (*relA spoT*) and assayed the dynamics of antibiotic tolerance seen in the experiment described for Fig. 2 with minor modifications (see Materials and Methods).

The ability of ϕ80 carriage to cause the same drop in persistence that we had previously attributed to the deletion of ten TA modules was worrying, because we had found this prophage in nearly all mutant strains that supported the model of persister formation proposed by our previous work (8). We therefore constructed new, uninfected versions of all mutants that had been found to be ϕ80 lysogens and assayed the dynamics of persister formation of these strains in order to determine whether the model still held (see Fig. 6A for a summary of the model and Materials and Methods for strain construction).

This model is based on the second messenger (p)ppGpp that plays important roles in persister formation of diverse organisms (2, 32). Consistently, we had previously reported severe defects in the formation or survival of ampicillin- and ciprofloxacin-tolerant persisters for mutants deficient in (p)ppGpp synthesis (*relA spoT* strains) (8). Subsequently, there had been some debate in the field whether pleiotropic phenotypes of these mutants such as slow growth, long lag times, and reduced stationary-phase viability might have affected our results (5, 16). We therefore compared the dynamics of antibiotic tolerance in cultures of a *relA spoT* mutant of *E. coli* K-12 MG1655 to those of the parental wild-type strain in a variant of our M9 medium that had been supplemented with a mixture of amino acids to support growth at roughly the same rate for the two strains (see Materials and Methods as well as the report by Potrykus et al. [33]). Under these conditions, we observed a considerable defect of the *relA spoT* mutant in tolerance to ciprofloxacin and gentamicin throughout all growth phases, though the overall shape of the curves following the levels of tolerant cells over time was only poorly affected (Fig. 6B and C). The *relA spoT* mutant also seemed to display a defect in tolerance to ampicillin during exponential growth, though the very different final cell densities and the early onset of full ampicillin tolerance upon cessation of growth by the *relA spoT* mutant made it difficult to judge this phenotype (Fig. 6B and C).

**FIG 7:**
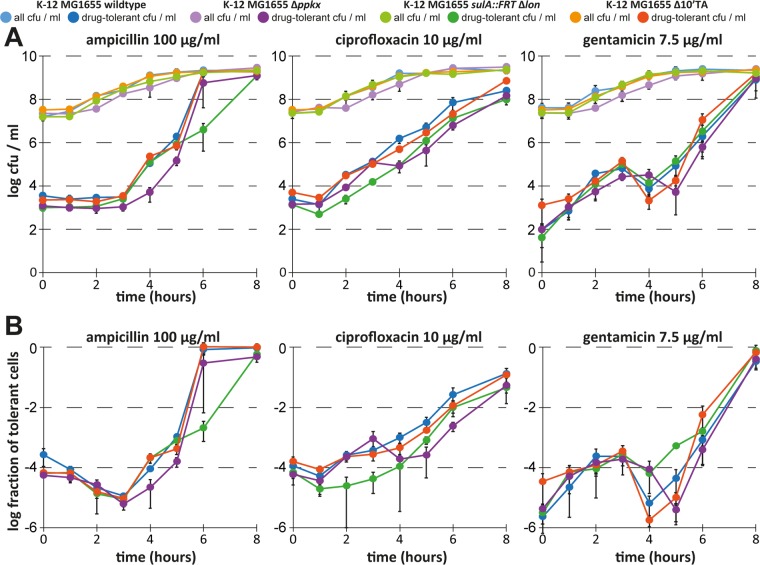
Protease Lon, but not mRNA endonuclease TA modules, contributes to persistence. We studied the dynamics of antibiotic tolerance for cultures of newly constructed *E. coli* K-12 Δ*ppkx*, *sulA*::*FRT* Δ*lon*, and *Δ10*′*TA* mutants in comparison to the parental wild type similarly to the experiment whose results are shown in Fig. 2. Changes in overall and antibiotic-tolerant CFU per milliliter are plotted in panel A, and the fractions of antibiotic-tolerant cells are plotted in panel B. While the *sulA*::*FRT* Δ*lon* mutant showed a clear defect in persister formation or survival during exponential growth (around 3 h after inoculation; see also Fig. S2), the parental *sulA*::*FRT* strain had no such phenotype (Fig. S3), confirming that the defect was due to the lack of Lon.

Downstream of (p)ppGpp, the model proposed by Maisonneuve et al. (8) comprises the production of polyphosphate by polyphosphate kinase (PPK) and degradation by exopolyphosphatase (PPX) (both lacking in the Δ*ppkx* deletion mutant that produces little polyphosphate), the polyphosphate-dependent activation of Lon (impaired in a *lon* deletion mutant), and the degradation of TA module antitoxins by Lon to activate toxins and induce persistence (impaired in a *Δ10TA* knockout mutant; Fig. 6A). Notably, the only one of these mutant strains of our previous studies that showed a defect in bacterial persistence but that had not been infected with ϕ80 was the *lon*::*tet* strain (Fig. 4D) (8, 9). However, the use of *lon* single mutants is prone to artifacts in persister assays due to the activation of SulA, an inhibitor of cell division, in response to DNA damage which is essentially irreversible in the absence of Lon, which degrades SulA (27, 34). We therefore created a *sulA lon* double mutant similar to the one that was used by Theodore et al. (34) in order to exclude these artifacts. This mutant showed a defect in the formation or survival of ciprofloxacin-tolerant persisters during exponential growth and also exhibited a markedly slower increase in ampicillin tolerance during stationary phase (Fig. 7). Conversely, the *ppkx* mutant showed a drop in ciprofloxacin tolerance only in stationary phase and was, beyond a slightly shifted curve likely caused by its slower growth, not affected in ampicillin tolerance (Fig. 7). We therefore conclude that our results confirm roles of (p)ppGpp as well as the Lon protease in the formation of persister cells by *E. coli* K-12, but these data cannot verify whether or not they are part of the same pathway as suggested previously. Furthermore, we failed to detect any phenotype of a newly constructed *Δ10TA* strain (called strain *Δ10*′*TA*) without prophages in tolerance to any of the tested antibiotics at any time point compared to the parental wild-type strain (Fig. 7). It appears therefore that unnoticed lysogenization with ϕ80 has been the reason for all of the persister phenotypes that we detected with the initial *Δ10TA* strain.

10.1128/mBio.01964-17.3 FIG S3  No phenotype of *sulA*::*FRT* in antibiotic tolerance. (A and B) As a control for the experiments performed with the *E. coli* K-12 MG1655 *sulA*::*FRT* Δ*lon* strain shown in Fig. 7, we studied the dynamics of antibiotic tolerance of the parental *sulA*::*FRT* mutant and plotted CFU per milliliter (A) as well as the fraction of antibiotic-tolerant cells (B). As expected from the literature, the *sulA* mutant strain showed no defect in antibiotic tolerance (see the study by A. Theodore, K. Lewis, and M. Vulicć, Genetics **195:**1265–1276, 2013, doi: 10.1534/genetics.113.152306. Data points show means of results from three independent experiments, and error bars represent standard deviations. Download FIG S3, PDF file, 0.5 MB. Copyright © 2017 Harms et al. 2017 Harms et al. This content is distributed under the terms of the Creative Commons Attribution 4.0 International license.

## DISCUSSION

### Persister phenotypes as an “inside job” of ϕ80.

In this study, we showed that important aspects of our previously published model of persister formation were based on the misinterpretation of experimental results caused by the unnoticed lysogenization of many of our strains with bacteriophage ϕ80. Consequently, defects in persister formation that we had previously attributed to different genetic mutations were instead a consequence of ϕ80 prophages residing in the chromosomes of these strains.

Others had previously questioned our model of persister formation based on a number of different considerations. Most importantly, some studies did not observe phenotypes of strains deficient in important components of our model in single-growth-time-point persister assays under their conditions. For example, Shan et al. (5) did not detect a defect of *lon sulA* and *ppkx* strains in persister formation or survival upon treatment with ampicillin and ciprofloxacin. Also, while the role of (p)ppGpp as an important controller of persister formation is generally well established (2, 32), recent studies suggested that the link between (p)ppGpp signaling and the activation of TA modules needs to be reinvestigated. As an example, an article by Ramisetty et al. (18) and an associated commentary by Van Melderen and Wood (13) raised the point that some of the antitoxins of the ten TA modules implicated in our model may not be targets of the Lon protease. Furthermore, they used the *yefM*-*yoeB* TA module as a model to study the link between (p)ppGpp and TA module activation and reported that polyphosphate was, in their hands, not required for *yefM*-*yoeB* activation after experimental induction of (p)ppGpp signaling or Lon overexpression (18). We agree that the role of polyphosphate requires further investigation, and, with the refined methodology of our current study, we could not confirm a role of this molecule in the formation and survival of persister cells during exponential growth of *E. coli* K-12 (Fig. 7). For Lon, others had previously argued that our failure to use a *sulA lon* double mutant might be the reason that we detected persister phenotypes of Lon-deficient strains that they were unable to reproduce (5, 34). In our hands, the *sulA lon* double mutant showed a clear 1-log reduction in the levels of ciprofloxacin-tolerant persisters during exponential growth (Fig. 7), supporting our previous observations (8) and suggesting that the degradation of Lon targets—either specific targets like antitoxins or simply unfolded proteins—is important for persister formation or survival. Future studies should therefore further investigate the links between (p)ppGpp, protease Lon, and bacterial persistence.

Beyond Lon and polyphosphate, considerable criticism revolved around the issue of whether our *Δ10TA* strain was a useful tool to study the link between TA modules and bacterial persistence. Others have reported various signs of slightly decreased viability of the original *Δ10TA* strain, such as a minor drop in the MIC of several antibiotics, that they traced back to polar effects of our TA module deletions or of additional mutations acquired during the long series of recombineering steps (13, 18). Notably, changes in MIC should not affect persistence *per se* (19), but it is entirely possible that these phenotypes of the *Δ10TA* strain are caused by the ϕ80 prophage that indeed increases the sensitivity of *E. coli* K-12 to, e.g., DNA-damaging agents (30). Beyond ampicillin and ciprofloxacin, we systematically included in this study the aminoglycoside gentamicin in our antibiotic killing assays but did not detect any relevant differences in tolerance to this antibiotic (apart from the results seen with the *relA spoT* mutant; Fig. 6). We chose gentamicin as a control because others showed previously that persister cells formed through the activity of mRNA endonuclease toxins should not be appreciably tolerant to aminoglycoside antibiotics, since these toxins fail to fully shut down translation and thus cannot prevent its poisoning by aminoglycoside drugs (35). Notably, other mechanisms of persister formation such as the activation of different TA modules (with small peptide toxins that abrogate the proton motive force) readily confer tolerance to aminoglycosides (4).

### Current evidence for a role of TA modules in persister formation.

Though we conclude that no evidence remains to support a role of the set of 10 mRNA endonuclease toxin TA modules in persister formation of *E. coli* K-12 in unstressed cells, we continue to favor the hypothesis that at least some of these elusive genetic elements act as phenotypic switches into dormancy, a view largely shared by others in the field (1, 2, 32, 36). Others have confirmed important roles of different sets of TA modules in persister formation of *E. coli* K-12 and related organisms such as uropathogenic *E. coli* and *Salmonella* (3, 4, 17, 37). With respect to mRNA endonuclease TA modules, phenotypes of even single TA module deletions under specific conditions have repeatedly been described in the field; e.g., *yafQ* mutants of *E. coli* K-12 were found to be specifically defective in persister formation inside bacterial biofilms (38–40). Consistently, it is becoming clearer and clearer that different TA modules in *E. coli* and beyond may respond to different forms of upstream signaling (5, 41). We therefore argue that the role of mRNA endonuclease TA modules in persister formation of *E. coli* K-12 and a possible link to Lon deserve further investigation, even though our previously reported phenotypes with TA modules do not hold true in the absence of ϕ80.

### Raising awareness of bacteriophage ϕ80.

Beyond the confusion created by our erroneous interpretation of results obtained with ϕ80-infected strains, we are worried that we have sent these strains to many research groups. Though it has been one of the favorite model phages in the early days of molecular biology (29), ϕ80 is now notorious as a laboratory contaminant. This bacteriophage combines “opportunistic induction” (i.e., a high frequency of spontaneous lytic development) with “stealth infectivity," i.e., the comparably low infectivity of particles at 37°C and a relatively high preference for lysogeny (30, 31). Contamination of strains with ϕ80 can therefore go unnoticed for a long time—*E. coli* K-12 *Δ5TA*, the first strain of the TA module deletion series carrying ϕ80, was first published by Christensen et al. in 2004 (42). However, elusive phenotypes such as a reduced efficiency of P1*vir* transductions can represent strong hints of ϕ80 contaminations (30). Due to the inability of ϕ80 to adsorb to stationary-phase cells, overnight cultures of lysogens regularly build up very high titers without any apparent sign of lysis (see also Fig. 5C), facilitating contamination. Additionally, ϕ80 readily spreads through P1*vir* transductions from infected strains (30). We can therefore only suggest that our colleagues regard the danger of ϕ80 contamination seriously and use simple PCR tests such as the one represented by Fig. 4B to unravel if enigmatic phenotypes may be caused by ϕ80 infection. Of note, we have tested a random sample of (published) *E. coli* strains that we had obtained from different laboratories at different times and found several independent cases of ϕ80 infection.

### A refined methodology to study bacterial persister formation.

Beyond the effects of ϕ80, we showed that the use of intermediate concentrations of fluoroquinolones (such 0.5 to 1 µg/ml of ciprofloxacin) for *E. coli* persister assays is inappropriate, because—in full congruency with the results of Sandvik et al. (26) for *S. aureus*—ca. 1 log of bacterial killing at these concentrations is caused not directly by the antibiotic but rather by the induction of resident prophages (Fig. 2). It therefore cannot be determined with certainty if phenotypes observed with experimental interventions or mutant strains are caused by differences in persister formation/survival or by effects on prophage induction. Future studies should therefore use higher concentrations of these antibiotics that inhibit prophage development, e.g., 10 µg/ml of ciprofloxacin.

Our results further suggest that protocols commonly used to study “exponential-phase” persister formation are greatly affected by the carryover of dormant cells and/or molecules from overnight cultures (Fig. 1 and 2). This effect may mask actual exponential-phase persister formation until the absolute level of newly formed persister cells overcomes the level of inoculated dormant cells. It seems likely to us that this phenomenon may have contributed to the inconsistencies of results published by different laboratories, because persister assays performed with antibiotic treatment at the same cell densities can give very different results depending on the amount of the initial inoculum (Fig. 1). Future studies should consider this effect in designing experiments and, if possible, should, e.g., include controls of bacteria that are in a state of balanced growth, i.e., that have completed an amount of divisions since their inoculation from stationary phase sufficient for effects of carryover to be excluded (43).

In this study, we regularly used a very laborious setup of antibiotic killing assays that followed the levels of antibiotic-tolerant CFU per milliliter throughout the different growth phases of *E. coli* (see Fig. 2A). Similar experiments have already been performed previously, e.g., in the seminal study by Keren et al. (44). Though a number of experimental parameters differed between that study and our current work, there is clear agreement that the levels of β-lactam and fluoroquinolone tolerance are similar in early growth phases after inoculation and later increase strongly until they reach (for β-lactams) full tolerance when the bacteria cease growth (Fig. 1 and 2; compare the results reported by Keren et al. [44]). It seems likely that the stable level of tolerant cells after inoculation may reflect a long lag phase of dormant cells carried over from stationary phase, and others have indeed found growth lag to be the most important aspect of antibiotic tolerance for bacteria that are inoculated from stationary phase directly into fresh medium with antibiotics (45). It has therefore already been reasonably argued in the field that future studies should follow sufficiently elaborate methodologies in order to unravel the complexity of various antibiotic-tolerant subpopulations in bacterial cultures (16, 19, 21). We have also observed very interesting and highly divergent dynamics of *E. coli* K-12 tolerance to different antibiotics and in different media (Fig. 1 and 2) that could not be explored in this study. However, it seems a promising field for future studies to compare the appearance and disappearance of tolerant cells for different antibiotics and for trying to uncover which external and intrinsic factors control antibiotic tolerance.

## MATERIALS AND METHODS

### Bacterial strains and their construction.

*E. coli* mutants were routinely constructed using recombineering with expression of the lambda red recombination genes from plasmid pWRG99 (46, 47). Clean deletions were constructed in a two-step procedure that first replaced the target gene(s) with the double-selectable cassette of template plasmid pWRG100, conferring chloramphenicol resistance for positive selection and carrying an I-SceI site for negative selection upon expression of the I-SceI endonuclease from pWRG99 (47). The double-selectable cassette was then removed through recombineering with a pair of annealed 80mer oligonucleotides spanning the desired deletion site with 40-bp homologies on each side. All bacterial strains used in this study are listed in Table S1 in the supplemental material. The nucleotide sequences of all oligonucleotide primers are listed in Table S2.

10.1128/mBio.01964-17.5 TABLE S1  List of all bacterial strains. This table lists the genotypes of all bacterial strains used in this study and additional information regarding construction or source. Download TABLE S1, PDF file, 0.6 MB. Copyright © 2017 Harms et al. 2017 Harms et al. This content is distributed under the terms of the Creative Commons Attribution 4.0 International license.

10.1128/mBio.01964-17.6 TABLE S2  List of all oligonucleotides. This table lists the sequences of all oligonucleotide primers used in this study. Download TABLE S2, PDF file, 0.3 MB. Copyright © 2017 Harms et al. 2017 Harms et al. This content is distributed under the terms of the Creative Commons Attribution 4.0 International license.

*E. coli* K-12 MG1655 *relA*::*FRT spoT*::*cat*(*207*), also known as PDC47, was constructed in two steps. The *relA* gene was deleted by P1*vir* transduction of the *relA*::*kanR* allele of the corresponding deletion strain of the Keio collection and subsequent recombination of the flanking FLP recombination target (FRT) sites using pCP20 (46, 48). In a second step, the *spoT*::*cat*(*207*) allele was transduced from *E. coli* CF1693 (49).

*E. coli* K-12 MG1655 Δ*ppkx*, also known as AHK062, was constructed by two-step recombineering as described with amplification of the double-selectable cassette using prAH1469/prAH1470 and 80mer oligonucleotides prAH1491/prAH1492.

*E. coli* K-12 MG1655 *sulA*::*FRT* Δ*lon*, also known as AHK173, was constructed from the *E. coli* K-12 MG1655 *sulA*::*kanR* strain described by Maisonneuve et al. (8) in two steps. We first removed the kanamycin resistance cassette by recombination of the flanking FRT sites using pCP20 (46) and then deleted *lon* by two-step recombineering with a double-selectable cassette amplified using prAH1465/prAH1466 followed by recombineering with 80mer oligonucleotides prAH1493/prAH1494.

*E. coli* K-12 MG1655 *Δ10*′*TA*, i.e., *E. coli* K-12 MG1655 Δ*hicAB*::*FRT* Δ*mqsR*::*FRT* Δ*yafO*::*FRT* Δ*yhaV*::*FRT* Δ*higB*::*FRT* Δ*yhaV-yoeB* Δ*dinJ-yafQ* Δ*relBE* Δ*chpBS* Δ*mazF* (also known as AHK250), was constructed by sequential deletion of the remaining 5 TA modules/TA module toxins on the basis of *E. coli* K-12 MG1655 *Δ5*′*TA* strain described by Maisonneuve et al. (9) that has the genotype Δ*hicAB*::*FRT* Δ*mqsR*::*FRT* Δ*yafO*::*FRT* Δ*yhaV*::*FRT* Δ*higB*::*FRT*. That strain had been created by deleting five mRNA interferase toxins in reverse order with respect to the TA module deletions of mutant *Δ10TA*. Of note, mutant *Δ5*′*TA* (unlike mutant *Δ10TA* or mutant *Δ5TA*) does not carry a lambda or ϕ80 prophage and does not show any difference from the parental wild-type strain in bacterial persister formation (see Fig. S4 in the supplemental material). The initial study by Maisonneuve et al. (9) had described a substantial defect of the *Δ5*′*TA* strain in bacterial persistence, but we were unable to reproduce this phenotype (Fig. S4), suggesting that the original work might have erroneously mixed up bacterial strains or inadvertently infected the clone used for experimentation with ϕ80. We deleted the *yefM-yoeB* locus in the *Δ5*′*TA* strain using a double-selectable cassette amplified with prAH1633/prAH1652 and the 80mer oligonucleotide pair prAH1653/prAH1654. The *dinJ-yafQ* TA module was deleted with a double-selectable cassette amplified using prAH1627/prAH1649 and subsequent recombineering of 80mer oligonucleotides prAH1659/prAH1660. Subsequently, the *relBE* module was deleted using a double-selectable cassette amplified with primers prAH1631/prAH1651 and the 80mer oligonucleotides prAH1655/prAH1656. We deleted the *chpBS* locus with a double-selectable cassette amplified using prAH1648/prAH1626 followed by recombineering of 80mer oligonucleotides prAH1661/prAH1662. Finally, the *mazF* toxin gene was deleted using a double-selectable cassette amplified with prAH1629/prAH1650 and the 80mer oligonucleotides prAH1657/prAH1658. We confirmed the successful introduction of all deletions in the *Δ10*′*TA* strain and also the parental *Δ5*′*TA* strain using diagnostic PCRs over the TA loci.

10.1128/mBio.01964-17.4 FIG S4  The *Δ5*′*TA* strain displays no phenotype in bacterial persistence. Exponentially growing cultures of *E. coli* K-12 MG1655, the *Δ5*′*TA* strain, or the *Δ10TA attB*(*+*) strain were challenged with 1 µg/ml ciprofloxacin for 5 h in LB medium, and the fraction of surviving persister cells was determined. Data points represent means of results from three independent experiments, and error bars indicate the standard deviations. Download FIG S4, PDF file, 0.4 MB. Copyright © 2017 Harms et al. 2017 Harms et al. This content is distributed under the terms of the Creative Commons Attribution 4.0 International license.

*E. coli* K-12 MG1655 *ϕ80*(*+*) was generated by lysogenization of the parental wild-type strain with ϕ80 of strain *Δ10TA attB*(*+*). In short, culture supernatant of *E. coli Δ10TA attB*(*+*) was prepared as described for the plaque assays outlined below and 100 µl was added to a culture of *E. coli* K-12 MG1655 at an optical density at 600 nm (OD_600_) of ca. 0.2. The culture was agitated at 30°C until lysis occurred, and survivors were plated on LB agar plates. After overnight incubation at 37°C, single colonies were tested for a K-12 MG1655 wild-type chromosomal background (by PCR on TA module loci) and carriage of only wild-type ϕ80 (by sensitivity to lambda *cI*_*b221*_).

### Curing of the lambda prophage from *E. coli* K-12 *Δ10TA.*

The original *E. coli Δ10TA* strain described by Maisonneuve et al. (9) was cured of its lambda prophage via a two-step P1*vir* transduction procedure. In short, the lambda prophage was first replaced with a temperature-sensitive λRED variant, and this prophage was subsequently cured by P1*vir* transduction of a native *gal-bio* region with an unoccupied *attB* integration site from the *E. coli* K-12 MG1655 wild-type strain.

Strain HME71 described by Sawitzke et al. (50) carries a temperature-sensitive λRED prophage and was cured for its Δ(*srlA*-*recA*)301::Tn*10* insertion by P1*vir* transduction of the native locus from the *E. coli* K-12 MG1655 wild-type strain in order to restore tetracycline sensitivity by removal of the resistance marker carried on the Tn*10* transposon. Transduction of the wild-type locus was selected as sorbitol prototrophy (Srl^+^) and verified by screening for a Rec^+^ phenotype and tetracycline sensitivity. Subsequently, the resulting strain (MAS889) was transduced back to tetracycline resistance with a P1*vir* lysate raised on strain MAS242 that carries a mini-Tn*10* inserted close to *galE* near the *attB* lambda attachment site, thus establishing a tetracycline resistance marker that is genetically linked to λRED. Transductant colonies were screened for temperature sensitivity (i.e., the presence of λRED). A P1*vir* lysate of the resulting strain, MAS902, was used to transduce tetracycline resistance into *E. coli Δ10TA* in order to replace the resident lambda prophage with λRED. Successful replacement was verified by screening for temperature sensitivity. Subsequently, the wild-type *gal-bio* region with an unoccupied *attB* site was transduced from *E. coli* K-12 MG155 by selection for galactose and biotin prototrophy (Gal^+^ Bio^+^) and loss of temperature sensitivity, creating strain *E. coli Δ10TA attB*(*+*). Loss of tetracycline resistance was verified and successful curing of lambda prophages was confirmed by the detection of an unoccupied *attB* site using overspanning PCR with primer pair prMASgal/prMASbio of Baek et al. (51). Different efforts to cure ϕ80 infections from lysogens have remained unsuccessful, as reported earlier by others (30).

### Preparation of culture media.

Luria-Bertani (LB) broth was prepared by dissolving 10 g of tryptone (catalog no. LP0042; Oxoid), 5 g of yeast extract (catalog no. LP0021; Oxoid), and 10 g of sodium chloride (catalog no. 27810.364; VWR Chemicals) per liter of Milli-Q H_2_O and sterilized by the use of an autoclave. M9 medium was prepared as “M9 minimal medium (standard)” according to the Cold Spring Harbor protocols (52) with modifications in the form of 1× M9 salts (stock solution prepared from 5× M9 salts [catalog no. M6030; Sigma-Aldrich]), supplemented with 50 µl of a 10 mg/ml FeSO_4_ solution (catalog no. F8048; Sigma), 0.4% (wt/vol) Bacto Casamino Acids (catalog no. 223020; BD Biosciences) (from 20% [wt/vol] sterile-filtered stock), 0.4% (wt/vol) d-glucose (catalog no. 101176K; VWR Chemicals) (from 40% [wt/vol] stock), 2 mM MgSO_4_ (catalog no. 25.165.292; VWR Chemicals), 1 µg/ml thiamine (catalog no. T1270; Sigma), and 100 µM CaCl_2_ (catalog no. 26.764.298; VWR Chemicals). For (p)ppGpp-deficient strain *E. coli* K-12 MG1655 *relA*::*FRT spoT*::*cat*(*207*), Casamino Acids in the M9 medium were replaced with 400 µg/ml of l-serine and 40 µg/ml of all other amino acids (Sigma) (all ≥98% or ≥98% purity) to support reasonable growth of this delicate strain as described by Potrykus et al. (33).

### Persister assays.

The presence of persister cells in bacterial cultures is usually detected using biphasic kill curves in which the addition of a bactericidal antibiotic is followed first by rapid killing of regular cells and then by a second, slower phase in which persister cells are killed (1, 2). Persister cells can then be quantified by comparing the levels of survivors obtained with, e.g., different mutant strains after a given time of antibiotic treatment. Experimental procedures similar to the one used here had also been described in our previous work and in reports of numerous studies by others in the field (5, 8, 9, 11). These studies showed that antibiotic treatment in LB medium and different minimal media for exponentially growing bacteria of ca. 1 to 5 × 10^8^ CFU/ml results in biphasic killing with merely persisters surviving after 5 h. Similarly, we demonstrated biphasic killing under these conditions for the M9 minimal medium that we used throughout this work (Fig. S2).

Overnight cultures were inoculated from single colonies into 3 ml of LB or M9 medium and grown for ca. 16 h in plastic culture tubes (catalog no. 62.515.006; Sarstedt; the lid was taped at the 13-ml mark with its lower end to ensure uniform aeration of replicates). For persister assays based on a single growth time point as described in Fig. 1A, overnight cultures were diluted at 1:100 back into LB or M9 medium in Erlenmeyer flasks and agitated at 37°C in a water bath shaker until they were in the mid-exponential-growth phase (ca. 1 × 10^8^ CFU/ml to 5 × 10^8^ CFU/ml; reached in our setup after ca. 2 to 2.5 h in LB medium or 2.5 to 3 h in M9 medium). At that point, cultures were treated with lethal concentrations of different antibiotics (100 µg/ml ampicillin, 1 or 10 µg/ml ciprofloxacin, or 7.5 µg/ml gentamicin) for 5 h in plastic culture tubes under conditions of rigorous agitation. In parallel, CFU counts per milliliter of the cultures were determined by plating serial dilutions on LB agar plates. After antibiotic treatment, bacterial pellets of 1.5-ml samples were washed once in 1 ml of sterile phosphate-buffered saline (PBS), resuspended in 100 µl of sterile PBS, and serially diluted in sterile PBS. Samples (10 µl) were spotted on LB agar plates to quantify antibiotic-tolerant survivors. Agar plates were incubated at 37°C for at least 24 h, and CFU counts per milliliter were determined from spots containing 10 to 100 bacterial colonies. The fraction of persister cells was calculated as the ratio of the CFU counts per milliliter after and before antibiotic treatment.

In order to study the dynamics of antibiotic-tolerant cells throughout the different growth phases of *E. coli* (as outlined in Fig. 2A), we performed an experiment as described above but adjusted the time between subculturing and antibiotic treatment from 0 h (i.e., direct inoculation into fresh medium containing antibiotic) to 8 h. For experiments with the (p)ppGpp-deficient strain *E. coli* K-12 MG1655 *relA*::*FRT spoT*::*cat*(*207*), the M9 growth medium was supplemented with an amino acid mixture instead of Casamino Acids (see above) and the experiment was extended to 10 h. Though the *relA spoT* mutant and *E. coli* K-12 wild-type strain have roughly the same growth rate in this medium, the final CFU count per milliliter of the (p)ppGpp-deficient mutant is significantly lower, which regularly results in experimental artifacts due to the appearance of suppressor mutants during overnight cultures (33). We therefore set up overnight cultures in LB medium (where this effect is much less pronounced) and adjusted the inoculum of both the mutant and wild-type strains to ca. 10^6^ CFU per milliliter in order to account for the roughly 3-fold-higher CFU per milliliter of wild-type overnight cultures.

### Genome sequencing and analysis.

Genomic DNA of the *E. coli* K-12 MG1655 wild-type strain as well as of its *Δ5TA*, *Δ8TA*, and *Δ10TA attB*(*+*) derivatives was isolated by phenol-chloroform extraction and sequenced by GATC Biotech to a read length of 51 bp using an Illumina HiSeq 2500 sequencing platform. Between 5 and 10 million paired end reads were obtained for each strain, resulting in an average 200-fold sequencing depth. Sequencing reads were assembled using the Velvet assembler (53) in both *de novo* and referenced modes, where the previously published genome of the *E. coli* K-12 MG1655 wild-type strain (GenBank accession no. U00096) served as a template for the latter. Suspected prophage infection was detected by assembling the raw contigs (around 20 per strain) to the published *E. coli* K-12 MG1655 genome sequence (GenBank accession no. U00096) and examining those not matching the reference by BLAST searches.

### Prophage detection by PCR.

Prophage carriage was detected by PCR with oligonucleotide primer pairs that amplified the two junctions between the *E. coli* K-12 chromosome and the prophage in the *gal-bio* region (lambda) and at the *yciI* locus (ϕ80). Bacteriophage lambda was detected using primer pairs prMASgal/prMASint (*gal-int*) and prMASb2/prMASbio (*b2-bio*) that have been used previously for this purpose (51). Bacteriophage ϕ80 was detected using primer pairs prAH1506/prAH1499 (*kch-int*) and prAH1500/prAH1521 (*pinL-tonB*). The nucleotide sequences of all oligonucleotide primers are listed in Table S2.

### Phage techniques.

Our phage work followed standard techniques that had already been used by others to study ϕ80 infections (30, 31). Plaque assays were performed by spotting lytic phages or correspondingly treated supernatants of *E. coli* cultures on LB plates overlaid with a top agar containing *E. coli* strains as indicated. Lytic phage stocks were produced by diffusion of phage particles into SMG buffer (0.1 M NaCl, 10 mM MgSO_4_, 0.05 M Tris [pH 7.5], 0.01% gelatin, Milli-Q H_2_O) from top agar plates with confluent plaques on *E. coli* K-12 MG1655. Supernatants of overnight cultures of suspected ϕ80 lysogens or control strains were separated from bacterial material by centrifugation and then diluted 1:10 into SMG buffer containing 10% chloroform. For plaque assays, LB agar plates in square format (12 cm by 12 cm) were overlaid with 7 ml of LB top agar (0.7% agarose) containing 100 µl of an overnight culture of the indicated *E. coli* strains. After the top agar had solidified, 5 µl of phage of supernatant stocks was spotted on the top. Plates were incubated at 30°C (to enable efficient plaque formation by ϕ80 [30]) overnight until plaque formation was visible. Sensitivity of bacterial strains to lambda *cI*_*b221*_ was assayed by evaluating the growth of bacterial streaks that crossed lines of phage stock on LB agar plates supplemented with 20 mM MgSO_4_ and 5 mM CaCl_2_. The ability of various strains to grow after contact with lambda *cI*_*b221*_ was evaluated visually after overnight incubation of agar plates at 37°C.

### Quantification and statistical analysis.

Experiments were usually analyzed by calculating means and standard deviations of results from at least three biological replicates. Detailed information for each experiment is provided in the figure legends.
