# Supplementary figures and images for: Prophages and Growth Dynamics Confound Experimental Results with Antibiotic-Tolerant Persister Cells
Source: mBio. 2017 Dec 12;8(6):e01964-17. doi: 10.1128/mBio.01964-17 (PMC5727415; doi:10.1128/mBio.01964-17)

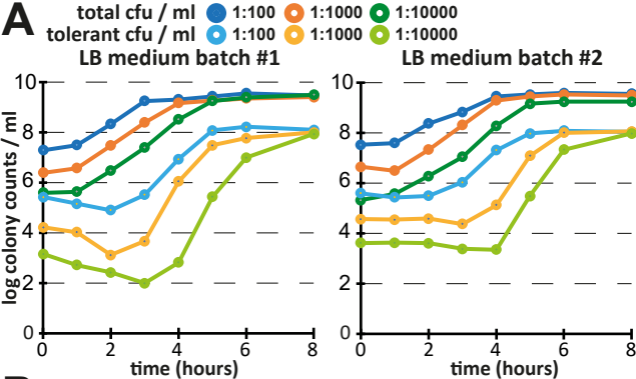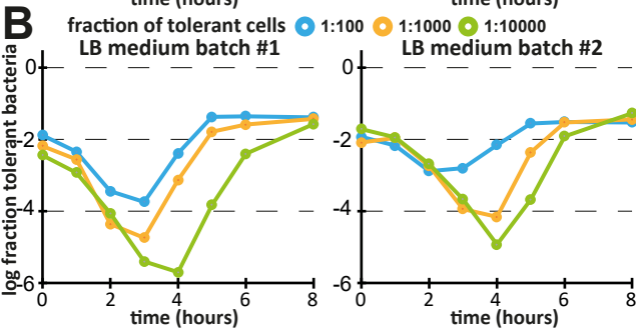

Supplement: FIG S1 [file mbo006173637sf1.pdf]

**A**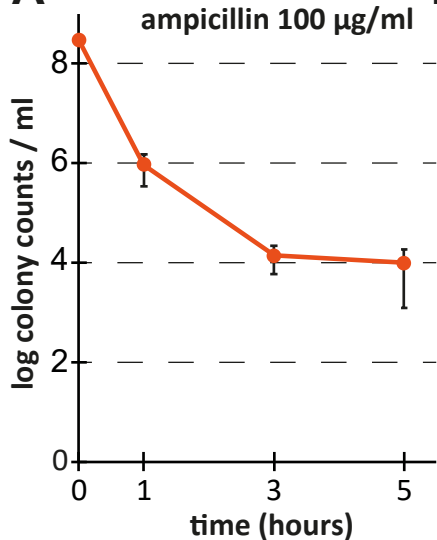**B**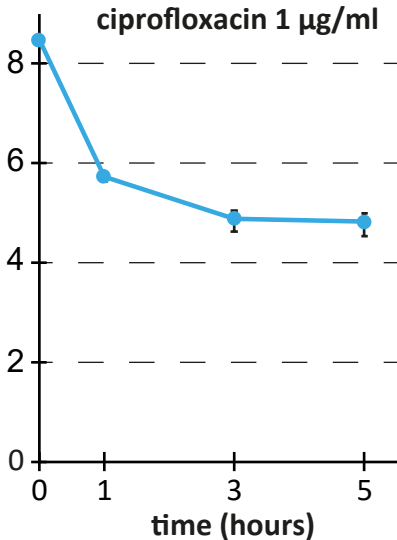**C**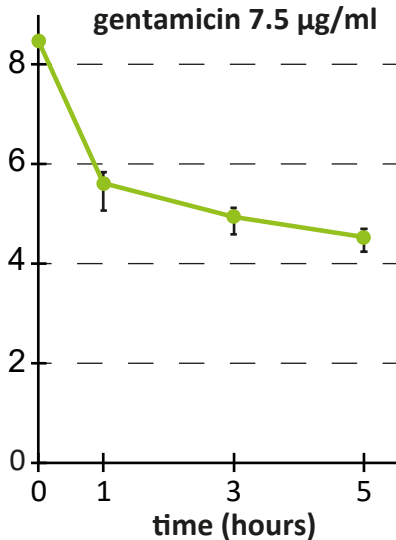

Supplement: FIG S2 [file mbo006173637sf2.pdf]

**A**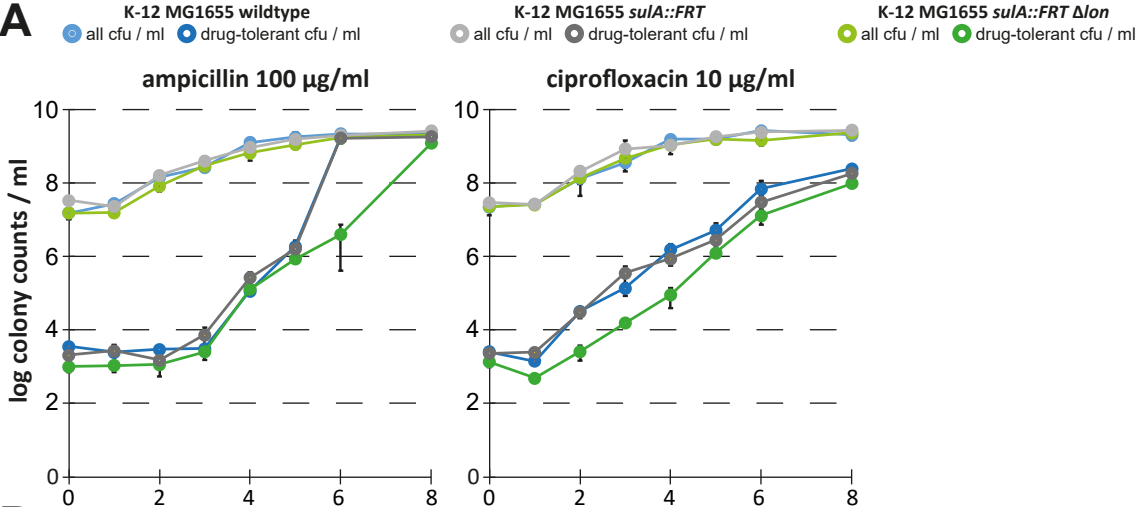**B**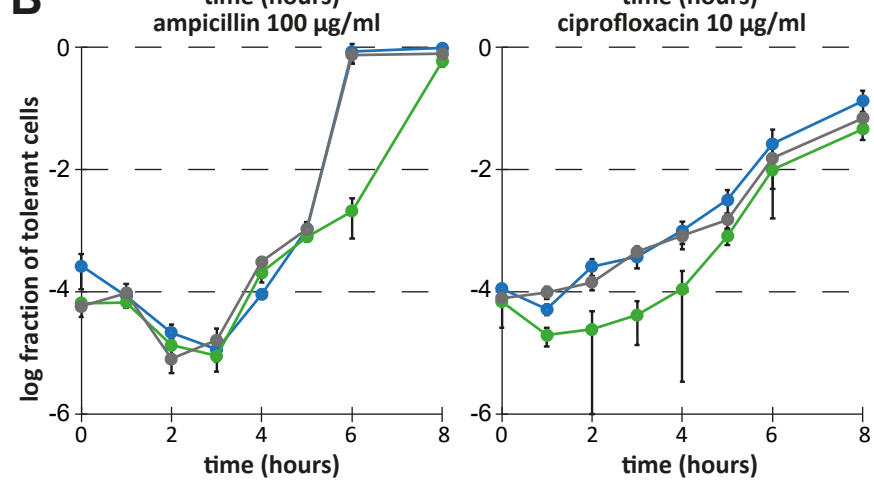

Supplement: FIG S3 [file mbo006173637sf3.pdf]

ciprofloxacin 1  $\mu\text{g/ml}$

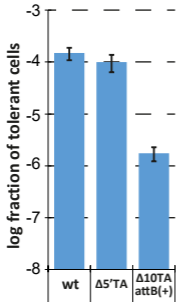

Supplement: FIG S4 [file mbo006173637sf4.pdf]
