# Supplementary material for: Prophages and Growth Dynamics Confound Experimental Results with Antibiotic-Tolerant Persister Cells
Source: mBio. 2017 Dec 12;8(6):e01964-17. doi: 10.1128/mBio.01964-17 (PMC5727415; doi:10.1128/mBio.01964-17)
Supplement: TABLE S1 [file mbo006173637st1.pdf]

Table S1. List of all strains used in this study.

Strain	Genotype*	Source / Description / Construction
E. coli K-12 MG1655	F^- λ^- *ilvG*$^-$ *rfb-50 rph-1*	our laboratory collection; K-12 wildtype strain
… Δ1TA	K-12 MG1655 Δ*chpB* ***λ_{def}(+)***	also known as SC31 (originally from S. K. Christensen, et al. (1)); used by E. Maisonneuve, et al. (2)
… Δ2TA	K-12 MG1655 Δ*mazF* Δ*chpB* ***λ_{def}(+)***	also known as SC301 (originally from S. K. Christensen, et al. (3)); used by E. Maisonneuve, et al. (2)
… Δ3TA	K-12 MG1655 Δ*mazF* Δ*chpB* Δ*relBE* ***λ_{def}(+)***	also known as SC3410 (originally from S. K. Christensen, et al. (3)); used by E. Maisonneuve, et al. (2)
… Δ4TA	K-12 MG1655 Δ*mazF* Δ*chpB* Δ*relBE* Δ*dinJ-yafQ* ***λ_{def}(+)***	also known as SC30146 (originally from S. K. Christensen, et al. (3)); used by E. Maisonneuve, et al. (2)
… Δ5TA	K-12 MG1655 Δ*mazF* Δ*chpB* Δ*relBE* Δ*dinJ-yafQ* Δ*yefM-yoeB* ***λ_{def}(+) φ80(+)***	also known as SC301467 (originally from S. K. Christensen, et al. (3)); used by E. Maisonneuve, et al. (2)
… Δ6TA	K-12 MG1655 Δ*mazF* Δ*chpB* Δ*relBE* Δ*dinJ-yafQ* Δ*yefM-yoeB* Δ*higBA* ***λ_{def}(+) φ80(+)***	also known as MGJ59; constructed and used by E. Maisonneuve, et al. (2)
… Δ7TA	K-12 MG1655 Δ*mazF* Δ*chpB* Δ*relBE* Δ*dinJ-yafQ* Δ*yefM-yoeB* Δ*higBA* Δ*prlF-yhaV* ***λ_{def}(+) φ80(+)***	also known as MGJ598; constructed and used by E. Maisonneuve, et al. (2)
… Δ8TA	K-12 MG1655 Δ*mazF* Δ*chpB* Δ*relBE* Δ*dinJ-yafQ* Δ*yefM-yoeB* Δ*higBA* Δ*prlF-yhaV* Δ*yafNO* ***λ_{def}(+) φ80(+)***	also known as MGJ5987; constructed and used by E. Maisonneuve, et al. (2)
… Δ9TA	K-12 MG1655 Δ*mazF* Δ*chpB* Δ*relBE* Δ*dinJ-yafQ* Δ*yefM-yoeB* Δ*higBA* Δ*prlF-yhaV* Δ*yafNO* Δ*mqsRA* ***λ_{def}(+) φ80(+) φ80h(80)imm(λ)(+)***	also known as MGJ59876; constructed and used by E. Maisonneuve, et al. (2)
… Δ10TA	K-12 MG1655 Δ*mazF* Δ*chpB* Δ*relBE* Δ*dinJ-yafQ* Δ*yefM-yoeB* Δ*higBA* Δ*prlF-yhaV* Δ*yafNO* Δ*mqsRA* Δ*hicAB* ***λ_{def}(+) φ80(+) φ80h(80)imm(λ)(+)***	also known as EJM43; constructed and used by E. Maisonneuve, et al. (2)
… Δ10 TA *attB(+)*	K-12 MG1655 Δ*mazF* Δ*chpB* Δ*relBE* Δ*dinJ-yafQ* Δ*yefM-yoeB* Δ*higBA* Δ*prlF-yhaV* Δ*yafNO* Δ*mqsRA* Δ*hicAB* ***φ80(+) φ80h(80)imm(λ)(+)***	this study; Δ10TA in which the defective lambda prophage had been cured (see *STAR Methods*)
… Δ5'TA	K-12 MG1655 Δ*hicAB::FRT* Δ*mqsR::FRT* Δ*yafO:::FRT* Δ*yhaV::FRT* Δ*higB::FRT*	also known as EJM46789; constructed and used by E. Maisonneuve, et al. (2)
… Δ10'TA	K-12 MG1655 Δ*hicAB::FRT* Δ*mqsR::FRT* Δ*yafO:::FRT* Δ*yhaV::FRT* Δ*higB::FRT* Δ*yefM-yoeB* Δ*dinJ-yafQ* Δ*relBE* Δ*chpBS* Δ*mazF*	this study; also known as AHK250
… λ(+)	K-12 MG1655 *lamB λ(+)*	also called JMT1; single lambda lysogen of S. Semsey, et al. (4)
… φ80(+)	K-12 MG1655 *ϕ80(+)*	this study; also called AHK031; K-12 MG1655 wildtype lysogenized with φ80 from culture supernatant of Δ10TA *attB(+)*
… *lamB*	K-12 MG1655 *lamB::camR*	obtained from Sine Lo Svenningsen
… *fhuA*	K-12 MG1655 *fhuA::kanR*	this study; *fhuA::kanR* allele transduced from the KEIO collection (5)
… *relA spoT* (original)	K-12 MG1655 *relA::kanR(251) spoT::: cat(207)* ***λ(+) φ80(+)***	also called CF1693 (originally from H. Xiao, et al. (6)); used by E. Maisonneuve, et al. (7) as stock EJM48; no indication that the lambda prophage is defective
… *relA spoT* (new)	K-12 MG1655 *relA::FRT spoT::cat(207)*	this study; also called PDC47
… *lon*	K-12 MG1655 *lon::tetR*	our laboratory collection; used by E. Maisonneuve, et al. (7)
… *sulA*	K-12 MG1655 *sulA::kanR*	constructed and used by E. Maisonneuve, et al. (7)
… *sulA* Δ*lon*	K-12 MG1655 *sulA::FRT* Δ*lon*	this study; also called AHK173
… Δ*ppk-ppx* (old)	K-12 MG1655 Δ*ppk ppx::kanR* ***φ80(+)***	also called CF5802 (originally from A. Kuroda, et al. (8)), used by E. Maisonneuve, et al. (7) as EJM47
… Δ*ppk-ppx* (new)	K-12 MG1655 Δ*ppk-ppx*	this study; also known as AHK062
BW25113	K-12 MG1655 F^- Δ*(araD-araB)567* Δ*lacZ4787(::rrnB-3)* λ^- *rph-1* Δ*(rhaD-rhaB)568 hsdR514*	our laboratory collection; commonly used lab strain derivative of K-12 MG1655
Δ9CP	BW25113 Δ*rac* Δ*CP4-57* Δ*CPS-53* Δ*DLP12* Δ*Qin* Δ*e14* Δ*CP4-6* Δ*CPZ-55*Δ*CP4-44* Δ*kanR*	derivative of BW25113 lacking all nine cryptic prophages; constructed by X. Wang, et al. (9)
HME71	W3110 *$galK_{tyr145UAG}$* Δ*lacU169* [λ c*I857* Δ(*cro-bioA*)] Δ(*srlA-recA*)301::*Tn*10	our laboratory collection; *E. coli* K-12 stain carrying the temperature-sensitive λRED prophage; constructed by J. A. Sawitzke, et al. (10)

MAS889	W3110 *galK*$_{tyr145UAG}$ Δ*lacU169* [λ cI*857* Δ(*cro-bioA*)] *srl*$^{+}$ *rec*$^{+}$ *tet*^{S}	this study; derivative of HME71 cured for the Δ(*srlA-recA*)301::*Tn*10) insertion
MAS242	K-12 MG1655 Δ(*proAB-lac*) *cys Ilv*(*val*^{R}) *str*^{R} *thi* mini-*Tn*10 close to *galE*	our laboratory collection
MAS902	W3110 *galK*$_{tyr145UAG}$ Δ*lacU169* [λ cI*857* Δ(*cro-bioA*)] *srl*$^{+}$ *rec*$^{+}$ *tet*^{S} *tn*10 close to *galE*	this study; *tn10* close to *galE* transduced from MAS242 into MAS889
**Bacteriophages**		
T4D	wildtype strain	obtained from Kenneth Kreutzer
φ80*vir*	obligately lytic mutant of φ80	obtained from Sine Lo Svenningsen
lambda *cI*$_{b221}$	obligately lytic mutant of lambda (*cI* mutant)	our laboratory collection

* newly discovered genotypic features of previously published strains are highlighted in bold font; the defective lambda prophage of some strains is indicated as λ_{def}

References cited in Table S1

1. **Christensen SK, Pedersen K, Hansen FG, Gerdes K.** 2003. Toxin-antitoxin loci as stress-response-elements: ChpAK/MazF and ChpBK cleave translated RNAs and are counteracted by tmRNA. J Mol Biol **332:**809-819.
2. **Maisonneuve E, Shakespeare LJ, Jørgensen MG, Gerdes K.** 2011. Bacterial persistence by RNA endonucleases. Proc Natl Acad Sci U S A **108:**13206-13211.
3. **Christensen SK, Maenhaut-Michel G, Mine N, Gottesman S, Gerdes K, Van Melderen L.** 2004. Overproduction of the Lon protease triggers inhibition of translation in *Escherichia coli*: involvement of the *yefM-yoeB* toxin-antitoxin system. Mol Microbiol **51:**1705-1717.
4. **Semsey S, Campion C, Mohamed A, Svenningsen SL.** 2015. How long can bacteriophage λ change its mind? Bacteriophage **5:**e1012930.
5. **Baba T, Ara T, Hasegawa M, Takai Y, Okumura Y, Baba M, Datsenko KA, Tomita M, Wanner BL, Mori H.** 2006. Construction of *Escherichia coli* K-12 in-frame, single-gene knockout mutants: the Keio collection. Mol Syst Biol **2:**2006 0008.
6. **Xiao H, Kalman M, Ikehara K, Zemel S, Glaser G, Cashel M.** 1991. Residual guanosine 3',5'-bispyrophosphate synthetic activity of *relA* null mutants can be eliminated by *spoT* null mutations. J Biol Chem **266:**5980-5990.

7. **Maisonneuve E, Castro-Camargo M, Gerdes K.** 2013. (p)ppGpp controls bacterial persistence by stochastic induction of toxin-antitoxin activity. Cell **154:**1140-1150.

8. **Kuroda A, Murphy H, Cashel M, Kornberg A.** 1997. Guanosine tetra- and pentaphosphate promote accumulation of inorganic polyphosphate in *Escherichia coli*. J Biol Chem **272:**21240-21243.

9. **Wang X, Kim Y, Ma Q, Hong SH, Pokusaeva K, Sturino JM, Wood TK.** 2010. Cryptic prophages help bacteria cope with adverse environments. Nat Commun **1:**147.

10. **Sawitzke JA, Thomason LC, Costantino N, Bubunenko M, Datta S, Court DL.** 2007. Recombineering: *in vivo* genetic engineering in *E. coli*, *S. enterica*, and beyond. Methods Enzymol **421:**171-199.
