# Supplementary material for: Prophages and Growth Dynamics Confound Experimental Results with Antibiotic-Tolerant Persister Cells
Source: mBio. 2017 Dec 12;8(6):e01964-17. doi: 10.1128/mBio.01964-17 (PMC5727415; doi:10.1128/mBio.01964-17)
Supplement: TABLE S2 [file mbo006173637st2.pdf]

Table S2. List of all oligonucleotide primers used in this study.

Primer name	*Sequence (5'-3')*
prAH1465	TCGTGTCATCTGATTACCTGGCGGAAATTAAACTAAGAGAGAGCTCTATGGGAACTTCATTTAAATGGCG
prAH1466	TTTTTATTAGTGCATTTTGCGCGAGGTCACTATTTTGCAGTCACAACCTGGGTCCATATGAATATCCTCCTTAG
prAH1469	ATAATATCCAGGCAGTGTCCCGTGAATAAAACGGAGTAAAAGTGGTAATGGGAACTTCATTTAAATGGCG
prAH1470	GCCGACATTTCTCGTCGGCCCGCAAAGTATTAAGCGGCGATTTCTGGTGTGGTCCATATGAATATCCTCCTTAG
prAH1491	GGCAGTGTCCCGTGAATAAAACGGAGTAAAAGTGGTAATGACACCAGAAATCGCCGCTTAATACTTTGCGGGCCGACGAG
prAH1492	CTCGTCGGCCCGCAAAGTATTAAGCGGCGATTTCTGGTGTCATTACCACTTTTACTCCGTTTTATTCACGGGACACTGCC
prAH1493	TGATTACCTGGCGGAAATTAAACTAAGAGAGAGCTCTATGCAGGTTGTGACTGCAAAATAGTGACCTCGCGCAAAATGCA
prAH1494	TGCATTTTGCGCGAGGTCACTATTTTGCAGTCACAACCTGCATAGAGCTCTCTCTTAGTTTAATTTCCGCCAGGTAATCA
prAH1499	CTTTGTGTCGGTGATCATTG
prAH1500	GAATAGCGAGCGTACCTTAG
prAH1506	GAAGTCTTTGCGCAGTATCAG
prAH1521	GGTCATTGAAGTCATAATCATTTC
prAH1626	TGCCCGCGATAAATAAACACACCTTATTCCACCACCGCCTGCAAGCGTAAGGTCCATATGAATATCCTCCTTAG
prAH1627	AGCGGGCTTTACCCAAAGAGCGCCGCGTGAGTTCCAGTTCTCTCAAATCGGGAACTTCATTTAAATGGCG
prAH1629	AGAATAGAAGTGAGTTAGTAACACTACCCAATCAGTACGTTAATTTTGGCGGAACTTCATTTAAATGGCG
prAH1631	AAACAGAGATGTCATGCTTTGGTTCAGAGAATGCGTTTGACCGCCTCGCTGGAACTTCATTTAAATGGCG
prAH1633	ATTCATTCTATGAATGAATCTGTTCAATAATGATAACGACATGCTGCAATGGAACTTCATTTAAATGGCG
prAH1648	TGTTTTGTAATTCCCTCACCTTTTGCTTTTCTCTCCGAGCCGCTTTCCATGGAACTTCATTTAAATGGCG
prAH1649	TCAATAATGGCTGGTCATGCTTTAATGTTGCAGTGTATCCCGGATCGTTTGGTCCATATGAATATCCTCCTTAG
prAH1650	TCCACGAGAATATCGACTGGGGAGAGCCGAAAGATAAGGAAGTCTGGTAAGGTCCATATGAATATCCTCCTTAG
prAH1651	CTTAGCAATGGCTGCTCCTGGCACAAAGCGGACAGTGATCACCGTTCTTAGGTCCATATGAATATCCTCCTTAG
prAH1652	CTGATGGCACCAAAAATAGCGAAGCCTGGATTTTCGTCTCACTGACAATAGGTCCATATGAATATCCTCCTTAG
prAH1653	TGAATGAATCTGTTCAATAATGATAACGACATGCTGCAATTATTGTCAGTGAGACGAAAATCCAGGCTTCGCTATTTTTG
prAH1654	CAAAAATAGCGAAGCCTGGATTTTCGTCTCACTGACAATAATTGCAGCATGTCGTTATCATTATTGAACAGATTCATTCA
prAH1655	GTCATGCTTTGGTTCAGAGAATGCGTTTGACCGCCTCGCTTAAGAACGGTGATCACTGTCCGCTTTGTGCCAGGAGCAGC
prAH1656	GCTGCTCCTGGCACAAAGCGGACAGTGATCACCGTTCTTAAGCGAGGCGGTCAAACGCATTCTCTGAACCAAAGCATGAC
prAH1657	TGAGTTAGTAACACTACCCAATCAGTACGTTAATTTTGGCTTACCAGACTTCCTTATCTTTCGGCTCTCCCCAGTCGATA
prAH1658	TATCGACTGGGGAGAGCCGAAAGATAAGGAAGTCTGGTAAGCCAAAATTAACGTACTGATTGGGTAGTGTTACTAACTCA
prAH1659	ACCCAAAGAGCGCCGCGTGAGTTCCAGTTCTCTCAAATCGAAACGATCCGGGATACACTGCAACATTAAAGCATGACCAG
prAH1660	CTGGTCATGCTTTAATGTTGCAGTGTATCCCGGATCGTTTCGATTTGAGAGAACTGGAACTCACGCGGCGCTCTTTGGGT

prAH1661	TTCCCTCACCTTTTGCTTTTCTCTCCGAGCCGCTTTCCATTTACGCTTGCAGGCGGTGGTGGAATAAGGTGTGTTTATTT
prAH1662	AAATAAACACACCTTATTCCACCACCGCCTGCAAGCGTAAATGGAAAGCGGCTCGGAGAGAAAAGCAAAAGGTGAGGGAA
prMASb2	GTATGCATGCTGGGTGTGGGGAA
prMASbio	CTCTCTGGCAAGCGCCTCGATT
prMASgal	CCGACAGAATCGGGCGAGAAGA
prMASint	CGCGCACGAAAAGCATCAGGT
